# Climatic patterns in the establishment of wintering areas by North American migratory birds

**DOI:** 10.1002/ece3.1973

**Published:** 2016-02-25

**Authors:** Heidi Pérez‐Moreno, Enrique Martínez‐Meyer, Jorge Soberón Mainero, Octavio Rojas‐Soto

**Affiliations:** ^1^ Red de Biología EvolutivaInstituto de Ecología A. C.XalapaVeracruzMéxico; ^2^ Departamento de ZoologíaInstituto de BiologíaUNAMMéxico D.FMéxico; ^3^ Natural History Museum and Biodiversity Research CenterDepartment of Evolutionary BiologyUniversity of KansasLawrenceKansas

**Keywords:** Climate, ecological niche models, migration, optimal areas, winter selection

## Abstract

Long‐distance migration in birds is relatively well studied in nature; however, one aspect of this phenomenon that remains poorly understood is the pattern of distribution presented by species during arrival to and establishment of wintering areas. Some studies suggest that the selection of areas in winter is somehow determined by climate, given its influence on both the distribution of bird species and their resources. We analyzed whether different migrant passerine species of North America present climatic preferences during arrival to and departure from their wintering areas. We used ecological niche modeling to generate monthly potential climatic distributions for 13 migratory bird species during the winter season by combining the locations recorded per month with four environmental layers. We calculated monthly coefficients of climate variation and then compared two GLM (generalized linear models), evaluated with the AIC (Akaike information criterion), to describe how these coefficients varied over the course of the season, as a measure of the patterns of establishment in the wintering areas. For 11 species, the sites show nonlinear patterns of variation in climatic preferences, with low coefficients of variation at the beginning and end of the season and higher values found in the intermediate months. The remaining two species analyzed showed a different climatic pattern of selective establishment of wintering areas, probably due to taxonomic discrepancy, which would affect their modeled winter distribution. Patterns of establishment of wintering areas in the species showed a climatic preference at the macroscale, suggesting that individuals of several species actively select wintering areas that meet specific climatic conditions. This probably gives them an advantage over the winter and during the return to breeding areas. As these areas become full of migrants, alternative suboptimal sites are occupied. Nonrandom winter area selection may also have consequences for the conservation of migratory bird species, particularly under a scenario of climate change.

## Introduction

A large body of research has focused on defining bird migration patterns between seasons, elucidating issues related to the evolution of migratory systems (Cox 1968, 1985; Levey and Stiles 1992; Rappole 1995; Chesser and Levey 1998; Zink 2002, 2011), geographical and ecological changes between seasons (Joseph and Stockwell 2000; Nakazawa et al. 2004) and migratory connectivity between breeding and wintering sites (Rubenstein et al. 2002; Webster et al. 2002; Somveille et al. 2015).

Birds are often thought to migrate in order to benefit from climatic seasonality that favors the seasonal availability of resources (H‐Acevedo and Currie 2003). However, there are other hypotheses for bird migration (Hurlbert and Haskell 2003; Somveille et al. 2015), and thus, the search for alternative but general explanations by which to understand this phenomenon continues. At a global scale, Somveille et al. (2013) found that strong spatial patterns emerge when patterns of diversity in migratory birds are pooled together, suggesting common underlying ecological drivers to which migratory birds respond. However, bird migration should be contextualized at different scales; for instance, there are several species within the tropics that migrate along altitudinal gradients, potentially following different ecological processes. In any case, different mechanisms related to different scales may not be mutually exclusive, given the dynamic nature of migration.

From this perspective, the diverse hypotheses proposed to explain bird migration, namely resource availability (MacArthur 1959; Newton and Dale 1996), seasonal productivity (Hurlbert and Haskell 2003; Dalby et al. 2014), competition with resident species (Rohwer et al. 2005), distance between breeding and nonbreeding ranges due to energetic costs (Wikelski et al. 2003), mortality (Newton 2008), and the avoidance of harsh winters and connectivity to breeding grounds (Somveille et al. 2015) could all be feasible explanations for diverse scales and groups of species. For instance, a clear bias is that most studies at the regional scale have focused on breeding migrants, with much less attention paid to the whereabouts of these species during the nonbreeding season (Somveille et al. 2015). This is an aspect that remains poorly understood, including the dynamics during migration periods, particularly in relation to the distribution patterns and their mechanisms within winter areas (Sillett and Holmes 2002).

It is assumed that wintering area selection is probably programmed by historical (i.e., evolutionary) factors, while the selection of sites within these wintering areas may be limited by biotic and abiotic factors (Cody 1985; Hutto 1985). At this level, and because migratory birds do not breed during the winter, access to food and reduction of predation risk appear to be the only selection criteria (Price 1981; Greenberg 1986); although evidence indicates that the abundance of migrants birds is also limited by factors affecting survival and physical condition during the nonbreeding season (Rappole and McDonald 1994; Sillett and Holmes 2002). However, as climate influences both the distribution of bird species and their resources, it is fair to conclude that the selection of winter areas is somehow determined by climate (Hutto 1985; Somveille et al. 2015).

Little research has focused on the effect of climate over the use of winter areas (Joseph 1996; Marra et al. 1998; Bearhop et al. 2004; Norris et al. 2004), but recent studies involving ENM (ecological niche modeling) have shown the critical importance of climatic conditions (the climatic niche) in the distribution patterns of species between wintering and breeding seasons (Nakazawa et al. 2004). Joseph (1996) and Nakazawa et al. (2004) distinguished three patterns: (1) where wintering climates are very different to climates in the breeding season (“niche shifters”), (2) where climates are very similar in both seasons (“niche followers”), and (3) intermediate cases (“mixed”). In this regard, and assuming a differential establishment of available sites, we hypothesized a selective establishment of areas during the winter, based on the ideal‐free distribution proposed by Fretwell (1972) and determined by the set of climatic conditions considered suitable for each species.

Research on specific competition during the winter has shown that the first individuals arriving to wintering ranges tend to occupy optimal sites (Morton 1976; Winker and Rappole 1992; Norris et al. 2004) and, because of the high turnover of individuals, such sites remain occupied all winter (Holmes et al. 1989; Stutchbury 1994). We therefore expect that, during the early winter months, individuals select and occupy sites that we assume are more favorable and which, at the macroscale, would be represented by those sites presenting optimal climates for the species, that is, “the hypothesis of selective establishment.” These sites may present similar climatic conditions and thus little variation. As the winter progresses, however, and the best sites are saturated (Rappole and Morton 1985), late migrant individuals must occupy suboptimal and marginal sites or adopt a “floating” strategy (Stutchbury et al. 2005; Brown and Long 2007; Sogge et al. 2007). This mechanism would produce a pattern in which climatic variation of occupied areas is low at the beginning and end of the wintering season, but peaks in the middle months of the season.

To test the selective establishment hypothesis, we analyzed the monthly climatic variation of the distribution of 13 Neotropical migratory birds throughout the winter season, using an ENM approach as a methodological tool with which to characterize the climatic niche of species (Soberón and Peterson 2005; Peterson et al. 2011). Understanding these and other factors of the biology of migration can help the development of appropriate conservation strategies, because winter conditions have proved to be of great importance in the life cycle of migrating species (Rappole et al. 1989; Rappole and McDonald 1994; Sherry and Holmes 1996; Marra et al. 1998).

## Materials and Methods

### Species selection and occurrence data

Following the American Ornithologists' Union (AOU), we sought for Neotropical migratory bird species with the following: (1) a winter distribution in Mexico and/or Central America, (2) a clear migration pattern (i.e., without overlap between the summer and winter ranges), and (3) a minimum sample size (September–April) of 10 spatially unique record points per month for model performance (Pearson et al. 2007). However, most species presented more than ten records per month (Table 1). Using a chi square test, we verified that sample‐size variation between months for each species did not affect the results (Appendix S1). Thirteen species of Passeriformes met these criteria and were therefore selected for analysis. According to the number of monthly records, niche models per month were generated for the period October to April for seven species, and from September to April for the other six (Table 1).

**Table 1:** Number of monthly unique occurrence records of 13 migratory bird species. The last column indicates the temporal span of occurrences and the number and percentage of occurrences before 1950. Species nomenclature follows the American Ornithologists' Union and further supplements

Species	Monthly records								Time span	Records before 1950	
	September	October	November	December	January	February	March	April		Number	%
*Cardellina pusilla*	122	158	137	178	226	201	244	105	1902–2009	130	9
*Oporornis tolmiei*		57	48	68	73	78	87	38	1882–2008	32	7
*Oreothlypis celata*	214	226	290	347	313	329	393	280	1904–2009	233	10
*Oreothlypis ruficapilla*	22	67	47	92	93	91	89	44	1891–2009	41	7
*Passerina ciris*		28	35	54	34	67	84	36	1891–2008	25	7
*Passerina cyanea*		61	63	66	88	111	140	70	1885–2009	39	6
*Piranga ludoviciana*		10	18	20	25	28	26	25	1895–2007	10	6
*Setophaga citrina*	10	12	15	12	20	15	24	11	1885–2008	11	9
*Setophaga magnolia*		21	23	18	23	28	36	36	1879–2009	17	9
*Setophaga nigrescens*	28	22	27	50	43	55	50	10	1887–2008	24	8
*Setophaga occidentalis*	14	11	11	19	17	13	16	10	1889–1999	5	4
*Setophaga virens*		11	15	25	22	14	18	12	1885–2008	11	9
*Spizella pallida*		13	28	16	11	16	28	18	1887–2002	7	5

**Figure 1:**
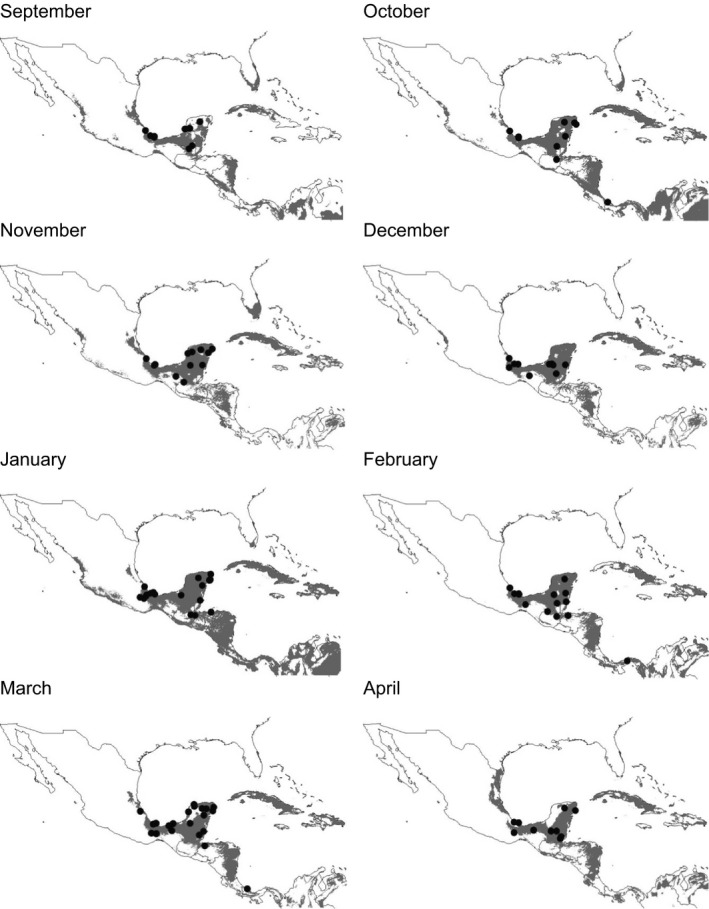
Example of monthly ecological niche models (*Setophaga citrina*). Monthly records appear as black points on each model.

Occurrence data were obtained from the Global Biodiversity Informatics Facility database (http://www.gbif.org/) and the Atlas of the Birds of Mexico (Navarro‐Sigüenza et al. 2002) with cross‐references made between these sources. Historical records lacking geographic coordinates but with location information were georeferenced with the database of locations of the National Institute of Statistics and Geography of Mexico (INEGI 2009). Regarding the spatial and temporal bias present in occurrence data, niche modeling methods correct for some of this bias because they extrapolate from samples of points to entire potential areas (Peterson et al. 2011) and potential temporal bias was considered by selecting only those species with statistically sufficient data points per month (Table 1, Appendix S1). The data in general were thus homogeneously distributed, both temporally (over the winter months of September to April and over the period 1879–2009: Table 1) and spatially (Fig. 1). Even though some temporal and spatial biases may remain, particularly in those months with low numbers of occurrences, the general patterns were clearly established for all species.

### Climatic characterization

As a source of climatic information, we used the WorldClim database (Hijmans et al. 2005). From this, we selected the three variables containing monthly averaged data from 1950 to 2000, including maximum and minimum temperatures (Tmax and Tmin) and precipitation (Prec) at a resolution of 0.0083 degrees (~1 km^2^). There may be concern regarding the temporal mismatch between occurrences and climate data; however, we consider that the WorldClim climatology, which spans over a 50‐year period (1950–2000), captures and represents the climatic variation for the last century, based on the knowledge that, on average, temperatures have warmed roughly 0.74°C over all land and ocean surfaces over this period and that more than half of this warming (about 0.4°C) has occurred since 1979 (IPCC 2007). Similar increases have been documented in precipitation during the last century (Hastenrath 2001). Moreover, the averaged data from the 1950 to 2000 layers actually represents the climatic period during which most of the occurrences take place and less than 8% of occurrences took place prior to 1950 (Table 1). The purpose of including historical data prior to 1950 was to complement the current distributional information, as suggested by Raxworthy et al. (2007).

### Ecological niche models

There are several algorithms for generating ecological niche models (Peterson et al. 2011). We chose the GARP (Genetic Algorithm for Production Rule, Stockwell and Noble 1992; Stockwell and Peters 1999), which is robust for low numbers of presence data points (Peterson 2001; Peterson et al. 2002; Tsoar et al. 2007). It has also proven effective when models need to be transferred to another scenario (e.g., Peterson et al. 1999; Joseph and Stockwell 2000; Peterson and Vieglais 2001; Rojas‐Soto et al. 2003; Nakazawa et al. 2004; Martínez‐Meyer and Peterson 2006; Martínez‐Meyer et al. 2006), as was the case in this study.

Genetic Algorithm for Production Rule includes inference methods that identify nonrandom associations between presence data and environmental variables in an iterative process of selection, evaluation, testing, and incorporation or rejection of a set of rules. To evaluate the predictive accuracy of the rules, GARP uses 1250 randomly selected nonpresence points throughout the study area to generate “pseudo‐absences” (Stockwell and Peters 1999). At each iteration, GARP selects the best rules, mixes them using operators that emulate evolutionary processes (mutations, deletions, and translocations), and generates a new set of predictive rules. Thus, an ecological niche model defined by GARP is a series of conditional rules (which are in the form of IF…THEN statements) used to determine whether the presence or absence of the species is predicted in a pixel (Peterson and Cohoon 1999), thus identifying portions of ecological space suitable for the species, which can be projected spatially in order to estimate its potential geographic distribution (Peterson 2001; Peterson et al. 2002; Tsoar et al. 2007). Models were validated via a chi‐square test using 20% of the occurrence data.

For each species, ecological niche models were performed for each month of the winter season (September to April). The result of each model was projected in geographic space and imported into a geographic information system (ArcView 3.2; ESRI 1999). Each monthly prediction (Fig. 1) was the result of superimposing the 10 best models (“best subset”) and selecting those areas where the 10 best models agreed (Anderson et al. 2003). Ten thousand pixels were randomly selected from the prediction area of each model, and their monthly climatic values were extracted. Finally, we obtained the CV (coefficient of variation) of each data set for each climate variable in each month.

### Monthly climatic variation coefficients and Akaike information criterion

Based on the hypothesis of selective establishment of wintering areas, the coefficients of variation of the three variables chosen should follow a parabolic shape, with low coefficients of variation for the first and last months of the season compared to the middle months. Thus, we generated 10 sets, each with 250 random points, based on the species winter distribution maps reported in the network “NatureServe,” which is available online (http://www.natureserve.org/) and considered null models as winter. We extracted monthly values of maximum temperature, minimum temperature, and precipitation for the selected points of the winter null model and calculated monthly coefficients of variation for each set of data. There are two questions: (1) Does the CV in variables follow a hump‐shaped distribution?, and (2) Are the values obtained by ENM a mere random sample of background environmental variability.

**Figure 2:**
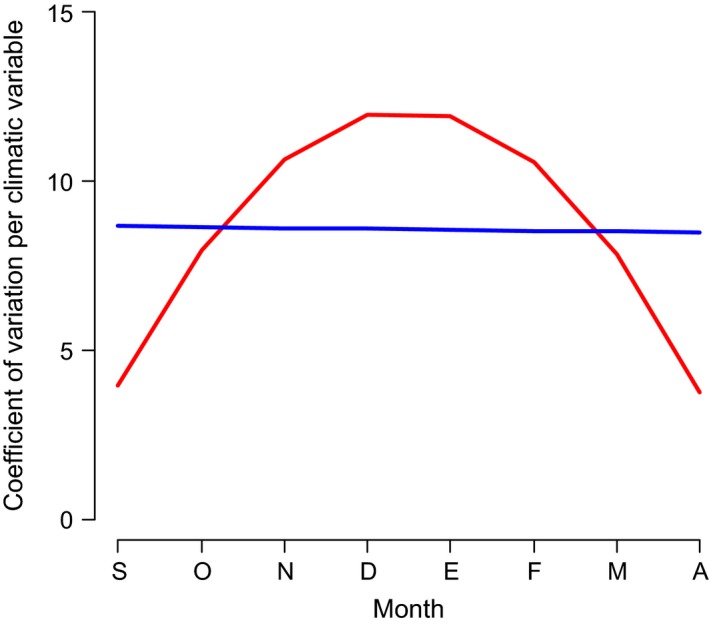
Quadratic model (red line) describing a parabolic pattern, indicating lower variation coefficients at the beginning and end of wintering season, and higher in the intermediate months. The linear model (blue line) assumes that coefficients of variation increase or decrease continually over the winter season.

To test the first question, we compared two GLM (generalized linear models), one quadratic and one linear, and used the AIC (Akaike information criterion) (Akaike 1973; Burnham and Anderson 2002; Symonds and Moussalli 2011) to evaluate whether the distribution of temperatures and precipitation CVs during the winter (obtained from the potential predictions of the winter months for each species) were described by the quadratic or the linear models (Fig. 2).

We calculated the delta (Δ_*i*_) and the Akaike weight (W_*i*_) in order to assess whether the differences among the candidate models were of sufficient magnitude to consider one as the best‐fit model (Burnham and Anderson 2002; Burnham et al. 2010). Akaike weight is a value between zero and one, and as the sum of the W_*i*_ of all models is one, the Akaike weight can be considered analogous to the probability that a given model is the best fit, which was considered as such when it was W_*i*_ **≥ 0.90 (Burnham and Anderson 2002). We used the R program and the package AICCmodAvg (Mazerolle 2015) to generate the GLM and calculate the values of AIC, delta, and Akaike weight.

To test the second question, that the values of the ENM are a simple random sample of the background (the wintering range according to published maps), for each of ten replicates, we fitted normal distributions to the variables in 250 random pixels. From these, we obtained one‐tailed probabilities for the observed CVs (niche model), assuming the null model distribution. Low values of probability of CV per variable, obtained from the ENMs of the winter months for each species, then indicate that the migratory birds select a site with a climatic variation lower than that of the background. In Table 2, we show the average value of probability for the ten replicates for each variable.

**Table 2:** Average value of probability of getting the observed coefficient of variation, per variable, in ten replicates of 250 random background points of the winter ranges reported for each species

Species	Tmax	Tmin	Prec
*Cardellina pusilla*	0.00342	0.05484	0.22779
*Oporornis tolmiei*	0.00069	3.76E‐08	0.0718
*Oreothlypis celata*	0.1712	0.03889	0.54055
*Oreothlypis ruficapilla*	0.06715	0.01428	0.125
*Passerina ciris*	0.00817	0.00006	0.00475
*Passerina cyanea*	0.00497	0.00028	0.01971
*Piranga ludoviciana*	0.00085	6.56E‐06	0.11956
*Setophaga citrina*	0.02419	0.00184	0.23135
*Setophaga magnolia*	0.02452	0.00253	0.06469
*Setophaga nigrescens*	0.01359	0.0959	0.07596
*Setophaga occidentalis*	0.13011	0.14102	0.22524
*Setophaga virens*	0.00018	2.16E‐13	0.14083
*Spizella pallida*	0.00009	5.27E‐07	0.25064

## Results

**Figure 3:**
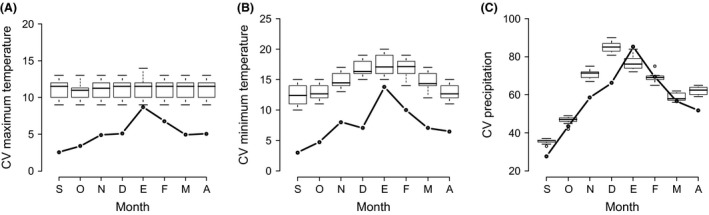
Distribution of winter climatic variability of a null model (box), respect to climate variation predicted by the ecological niche modeling (black line) for *Setophaga citrina*. Maximum temperature (A), minimum temperature (B), and precipitation (C). Temperatures have a low probability index (0.024 and 0.002) compared to precipitation (0.231).

A total of 97 models were obtained and the validation test showed that the majority of the models performed better than would be expected by chance (Appendix S2). The pattern of climatic variation of the niche models (measured throughout using the CVs of each monthly variable) is very different (lower) for the two temperatures (Fig. 3). On the other hand, in at least two of the three climatic variables used and for most of the species, variation of sites occupied during the winter was consistent with the hypothesis of selective establishment of areas (Table 2). Indeed, monthly ecological niche models for 11 of the 13 species showed that sites occupied during the early and late winter months were less variable than those occupied during the intermediate months (Appendices S3, S4).

**Figure 4:**
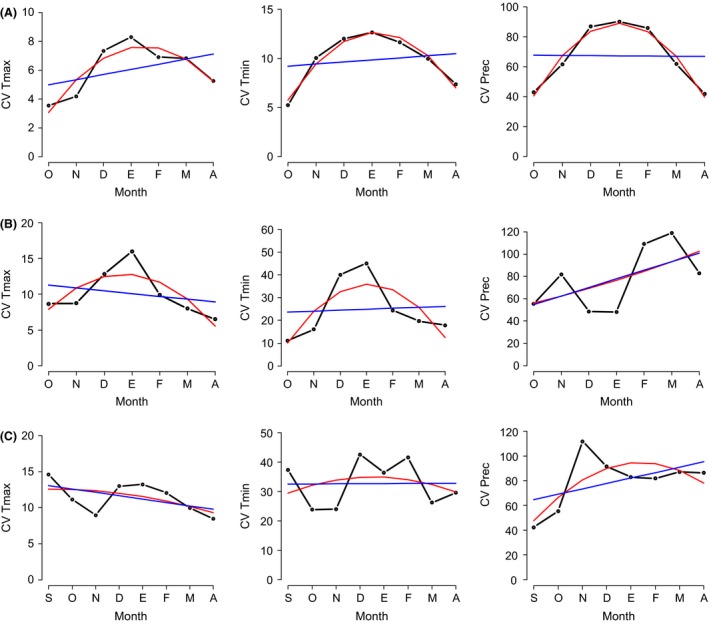
Distribution of monthly climatic variation (black line) of maximum and minimum temperature and precipitation during winter obtained from ecological niche models for *Setophaga magnolia* (A), *Piranga ludoviciana* (B), and *Oreothlypis ruficapilla* (C). The red and blue lines represent the expected distribution from the GLM‐derived, quadratic, and linear models, respectively.

In some species, such as *Passerina ciris, Passerina cyanea, Setophaga citrina*,* Setophaga magnolia,* and *Setophaga virens*, the distribution of monthly coefficients of variation for the three climatic variables was fitted to the quadratic model (Table 3, Fig. 4A, Appendix S3). This same pattern of occurrence (fitting the quadratic model) was exhibited by other species in two of the three macroclimatic variables; for example, the distribution of the coefficients of variation of maximum and minimum temperatures in *Piranga ludoviciana, Oreothlypis celata, Oporornis tolmiei,* and *Spizella pallida*, and minimum temperature and precipitation in *Setophaga nigrescens* and *Setophaga occidentalis* (Table 3, Fig. 4B, Appendix S4). On the other hand, and contrary to that predicted by the hypothesis of selective establishment, the distribution of monthly coefficients of variation of precipitation and temperatures in *Oreothlypis ruficapilla* (Table 3, Fig. 4C) and *Cardellina pusilla* did not fit the quadratic model (Table 3, Appendix S4).

**Table 3:** AIC (Akaike information criterion), delta (Δ_*i*_), and Akaike's weight (W_*i*_) values for the GLM analysis (quadratic and linear) those highlighted in bold agree significantly with the quadratic model. GLM were run for the monthly coefficient of variation for maximum and minimum temperature and precipitation in 13 migratory bird species

Variable	Models	Species	AIC	Δ_i_	W_i_	Species	AIC	Δ_i_	W_i_	Species	AIC	Δ_i_	W_i_
Tmax	Quadratic	*Cardellina pusilla*	28.7	0	0.2719	*Passerina cyanea*	20.1	0	**0.9989**	*Setophaga occidentalis*	43.3	0.32	0.4601
	Linear		26.8	−2	0.7281		33.8	13.7	0.0011		43	0	0.5399
Tmin	Quadratic		50.6	0	0.7640		38.1	0	**0.9904**		59.8	0	**0.9519**
	Linear		52.9	2.35	0.2360		47.4	9.28	0.0096		65.8	5.97	0.0481
Pre	Quadratic		67.3	0	**0.9923**		49.4	0	**0.9989**		76.3	0	**0.8952**
	Linear		77	9.71	0.0077		63	13.6	0.0011		80.6	4.29	0.1048
Tmax	Quadratic	*Oporornis tolmiei*	25.2	−4.8	**0.9161**	*Piranga ludoviciana*	35.7	0	**0.9276**	*Setophaga virens*	25.5	0	**0.9565**
	Linear		30	0	0.0839		40.8	5.1	0.0724		31.7	6.18	0.0435
Tmin	Quadratic		46.6	−4.3	**0.8971**		55.3	0	**0.9286**		33.6	0	**0.9361**
	Linear		51	0	0.1029		60.4	5.13	0.0714		39	5.37	0.0639
Pre	Quadratic		66.3	0	0.3775		70.9	1.98	0.2709		51.8	0	**0.9606**
	Linear		65.3	−1	0.6225		68.9	0	0.7291		58.1	6.39	0.0394
Tmax	Quadratic	*Oreothlypis celata*	44.3	0	**0.9762**	*Setophaga citrina*	30.1	0	**0.9309**	*Spizella pallida*	30.7	0	**0.9825**
	Linear		51.7	7.43	0.0238		35.3	5.2	0.0691		38.8	8.06	0.0175
Tmin	Quadratic		67.1	0	**0.9772**		39.2	0	**0.9481**		49.3	0	**0.9896**
	Linear		74.6	7.52	0.0228		45	5.81	0.0519		58.5	9.12	0.0104
Pre	Quadratic		68.4	0	0.2984		57.2	0	**0.9991**		58.1	1.94	0.2749
	Linear		66.7	−1.7	0.7016		71.2	14	0.0009		56.2	0	0.7251
Tmax	Quadratic	*Oreothlypis ruficapilla*	39.2	1.73	0.2963	*Setophaga magnolia*	21.2	0	**0.9930**				
	Linear		37.5	0	0.7037		31.2	9.93	0.0069				
Tmin	Quadratic		61.7	1.37	0.3351		15.5	0	**0.9999**				
	Linear		60.3	0	0.6649		38.4	22.9	0.0001				
Pre	Quadratic		72.4	0	0.7503		45.6	0	**0.9999**				
	Linear		74.6	2.2	0.2497		67.2	21.6	0.0001				
Tmax	Quadratic	*Passerina ciris*	21.1	0	**0.9885**	*Setophaga nigresens*	29.9	0	0.7301				
	Linear		30	8.91	0.0115		31.9	1.99	0.2699				
Tmin	Quadratic		36	0	**0.9157**		48.6	0	**0.9991**				
	Linear		40.8	4.77	0.0843		62.7	14.1	0.0009				
Pre	Quadratic		48.5	0	**0.9977**		67.2	0	**0.9133**				
	Linear		60.7	12.2	0.0023		71.9	4.71	0.0867				

## Discussion

Selection of wintering sites by migratory birds has been thought to be homogeneously distributed in geography, with variation produced by local factors or fine‐scale conditions such as resource availability, productivity, competition with resident species, the distance between breeding and nonbreeding ranges, connectivity, and the avoidance of harsh climatic conditions (e.g., MacArthur 1959; Newton and Dale 1996; Hurlbert and Haskell 2003; Wikelski et al. 2003; Rohwer et al. 2005; Newton 2008; Dalby et al. 2014; Somveille et al. 2015). Our results showed, however, that most species occupied areas under specific climatic conditions, as can be observed from the low climatic variation predicted by niche models with respect to null models.

The lower probability values of having the same distribution of CV as the background observed for the temperatures in all species analyzed, indicate that selection of wintering sites was based primarily on these variables, followed by precipitation (Table 2). Joseph (1996) showed that summer temperature was a determinant factor in the winter distributions of 92 migratory passerine species, while Joseph and Stockwell (2000) showed that the migration route of *Myiarchus swainson*, a southern migratory species, is determined by temperature, tracking as it does a specific thermocline. Furthermore, van Oudenhove et al. (2014) highlight how temperatures encountered throughout the annual cycle affect the vital rates of the greater snow goose (*Anser caerulescens*).

Although many studies have stressed the great influence of food resources on winter site selection (Salomonson and Balda 1977; Stutchbury 1994; Studds and Marra 2005; Townsend et al. 2010), our results suggest that climatic variables also play a highly important role in the selection of wintering areas (Joseph 1996; Marra et al. 1998; Bearhop et al. 2004; Nakazawa et al. 2004; Norris et al. 2004; van Oudenhove et al. 2014). Indeed, for most of the species studied, the selection of sites during the winter was not random with respect to climatic conditions, suggesting the existence of preferred winter areas (Hutto 1985) and differential establishment of these areas over time (Fretwell 1972).

The low numbers in the coefficients of variation for climatic variables at the beginning of the season suggest that the first individuals to arrive during the winter prefer certain climatic conditions, possibly because they have an advantage in acquiring better areas in winter (Morton 1976; Winker and Rappole 1992; Norris et al. 2004). Indeed, if molt is delayed during the summer, the departure to wintering areas is also delayed, thus reducing their chances to find optimal sites for wintering and minimizing the survival chances of individuals (Morton and Morton 1990). It is known, for instance, that many females of *S. citrina* do not produce a second brood during the summer due to the hidden costs incurred by both parent and offspring by arriving late in the fall and thus lowering the possibility of acquiring high‐quality sites during the winter (Evans‐Ogden and Stutchbury 1996).

Similarly, the low coefficients of climatic variation for the late winter months reported here suggest that suboptimal climates are emptied first while optimal sites remain occupied throughout the winter season. Indeed, the fidelity to sites during the winter that has been associated with favorable conditions (Brown et al. 2002; Somershoe et al. 2009; Latta and Faaborg 2011) suggests that occupants leave the optimal areas only during their spring migration. However, these individuals migrate before exhausting local resources, leaving those sites available for “floating” or subordinate birds (Salomonson and Balda 1977) that rapidly occupy these vacant territories in order to improve their physical condition before embarking upon the spring migration (Holmes et al. 1989; Stutchbury 1994; Marra et al. 1998).

Notwithstanding the previous results, we found two exceptions to the general selective establishment hypothesis pattern, namely *Cardellina pusilla* and *Oreothylpis ruficapilla*, but this may have been the result of taxonomic issues. For instance, recent studies suggest that breeding populations of these species may actually constitute independent lineages and therefore represent cryptic species (Kimura et al. 2002; Clegg et al. 2003; Ruiz‐Sánchez et al. 2015). This would incur confusion in any predictions regarding their winter distribution.

On the other hand, the ecologically restricted species might present lower variation during their winter season, and species such as *Oreothypis tolmiei, Piranga ludoviciana, Setophaga occidentalis*, and *Setophaga nigrescens* that inhabit mostly high elevations, or *Spizella pallida* that inhabits dry areas associated with thorn scrubs, would thus comprise the set of species that showed models that fitted two of the three climatic variables to the selective establishment hypothesis.

Looking at the pattern of establishment of wintering areas in each species in terms of distribution between seasons (Table 1), we found no clear pattern of “niche follower,” “switcher” or “mixed” (sensu Nakazawa et al. 2004). Therefore, we suggest that species select sites for wintering regardless of their breeding season climatic requirements. However, further studies are required analyzing a greater number of species in order to draw any firm conclusion in this regard.

In summary, we propose that the sequence and duration of establishment of wintering areas involves the selection of sites that favor the condition of the occupants during this season and affect the later stages of the annual cycle. This pattern of establishment of wintering areas provides advantages for individuals who are distributed in those areas with optimal climates for the species, and which generally achieve improved physical condition during the winter, migrate earlier in spring and subsequently present greater reproductive success and higher rates of return than individuals who spend the winter in climatically suboptimal areas (Marra et al. 1998; Bearhop et al. 2004; Norris et al. 2004).

Overall, our results provide evidence of the importance of climatic factors for understanding not only long‐distance migration, but also the distributional dynamics within wintering areas. This last point has been poorly addressed in the past, but has important implications for conservation given the decline in winter populations observed in many species of migratory birds as a result of habitat loss (Robbins et al. 1989; Askins et al. 1990). Our study shows that identification and prioritization of important wintering areas for conservation of migratory birds can be significantly enhanced by taking climatic requirements into consideration.

## Conflict of Interest

None declared.

## Supporting information


**Appendix S1.** Values of the Chi square test from a monthly comparison among the total number of occurrences per species (see Table 1 in the MS). No differences were found for 8 species using probability 0.05 (*) and for 11 species using probability 0.1 (**). For species with significant differences in the number of occurrences, the significant difference (value Z) between months is for one month only, and is generally the month with the highest number of occurrences.
**Appendix S2.** Significance of niche models using a Chi square test: *P* ≤ 0.001 (**), *P* ≤ 0.05 (*).
**Appendix S3.** Distribution of monthly climatic variation (black line) of maximum and minimum temperature and precipitation during winter obtained from ecological niche models (ENM) for species where three climatic variables fitted the quadratic model: *Passerina ciris, Passerina cyanea, Setophaga citrina,* and *Setophaga virens*. The red and blue lines represent the expected distribution from the GLM‐derived quadratic and linear models, respectively.
**Appendix S4.** Distribution of monthly climatic variation (black line) of maximum and minimum temperature and precipitation during winter obtained from ecological niche models (ENM) for species where two climatic variables fitted the quadratic model: *Cardellina pusilla, Oreothlypis celata, Oporornis tolmiei, Setophaga nigrescens, Setophaga occidentalis,* and *Spizella pallida*. The red line and blue line represent the expected distribution from the GLM‐derived quadratic and linear models, respectively.
