# Supplementary material for: Climatic patterns in the establishment of wintering areas by North American migratory birds
Source: Ecol Evol. 2016 Feb 25;6(7):2022–33. doi: 10.1002/ece3.1973 (PMC4831436; doi:10.1002/ece3.1973)
Supplement: Supplementary file 1 — Appendix S1. Values of the Chi square test from a monthly comparison among the total number of occurrences per species (see Table 1 in the MS). No differences were found for 8 species using probability 0.05 (*) and for 11 species using probability 0.1 (**). For species with significant differences in the number of occurrences, the significant difference (value Z) between months is for one month only, and is generally the month with the highest number of occurrences. Appendix S2. Significance of niche models using a Chi square test: P ≤ 0.001 (**), P ≤ 0.05 (*). Appendix S3. Distribution of monthly climatic variation (black line) of maximum and minimum temperature and precipitation during winter obtained from ecological niche models (ENM) for species where three climatic variables fitted the quadratic model: Passerina ciris, Passerina cyanea, Setophaga citrina, and Setophaga virens. The red and blue lines represent the expected distribution from the GLM‐derived quadratic and linear models, respectively. Appendix S4. Distribution of monthly climatic variation (black line) of maximum and minimum temperature and precipitation during winter obtained from ecological niche models (ENM) for species where two climatic variables fitted the quadratic model: Cardellina pusilla, Oreothlypis celata, Oporornis tolmiei, Setophaga nigrescens, Setophaga occidentalis, and Spizella pallida. The red line and blue line represent the expected distribution from the GLM‐derived quadratic and linear models, respectively. [file ECE3-6-2022-s001.docx]

**Supporting Information**

Additional Supporting Information may be found in the online version of this article.

**Appendix S1.** Values of the Chi square test from a monthly comparison among the total number of occurrences per species (see Table 1 in the MS). No differences were found for 8 species using probability 0.05 (*) and for 11 species using probability 0.1 (**). For species with significant differences in the number of occurrences, the significant difference (value Z) between months is for one month only, and is generally the month with the highest number of occurrences.

**Appendix S2.** Significance of niche models using a Chi square test: P ≤ 0.001 (**), P ≤ 0.05 (*).

**Appendix S3.** Distribution of monthly climatic variation (black line) of maximum and minimum temperature and precipitation during winter obtained from ecological niche models (ENM) for species where three climatic variables fitted the quadratic model: *Passerina ciris, Passerina cyanea, Setophaga citrina,* and *Setophaga virens*. The red and blue lines represent the expected distribution from the GLM-derived quadratic and linear models, respectively.

**Appendix S4.** Distribution of monthly climatic variation (black line) of maximum and minimum temperature and precipitation during winter obtained from ecological niche models (ENM) for species where two climatic variables fitted the quadratic model: *Cardellina pusilla, Oreothlypis celata, Oporornis tolmiei, Setophaga nigrescens, Setophaga occidentalis,* and *Spizella pallida*. The red line and blue line represent the expected distribution from the GLM-derived quadratic and linear models, respectively.

Appendix 1

| Specie | Chi^2^ | Pvalue | Monthly Differences |
| --- | --- | --- | --- |
| *Cardellina pusilla* | 101.44 | 5.44 E-19 |  |
| *Oporornis tolmiei* | 28.1 | 8.97 E-05 |  |
| *Oreothlypis celata* | 84.38 | 1.749 E-15 |  |
| *Oreothlypis ruficapilla* | 77.87 | 3.74 E -14 |  |
| *Passerina ciris* | 56.27 | 2.56 E-10 |  |
| *Passerina cyanea* | 62.56 | 1.35 E-11 |  |
| *Piranga ludoviciana* | 10.75 | 0.096 * | Oct (lowest) |
| *Setophaga citrina* | 11,084 | 0.135 * ** | Mar (highest) |
| *Setophaga magnolia* | 11.71 | 0.068 * | Mar-Apr (highest ) |
| *Setophaga nigrescens* | 51.02 | 9.07 E-09 |  |
| *Setophaga occidentalis* | 5.25 | 0.062 * | Dec (highest) |
| *Setophaga virens* | 9.77 | 0.13 * ** | Dec (highest) |
| *Spizella pallida* | 15.06 | 0.0197 |  |

Appendix 2

| Species | Sep | | | Oct | | | Nov | | | Dec | | | Jan | | | Feb | | | Mar | | | Apr | | |
| --- | --- | --- | --- | --- | --- | --- | --- | --- | --- | --- | --- | --- | --- | --- | --- | --- | --- | --- | --- | --- | --- | --- | --- | --- |
|  | Chi^2^ | | Pvalue | Chi^2^ | | Pvalue | Chi^2^ | | Pvalue | Chi^2^ | | Pvalue | Chi^2^ | | Pvalue | Chi^2^ | | Pvalue | Chi^2^ | | Pvalue | Chi^2^ | | Pvalue |
| *Cardellina pusilla* | 38.86 | 72.98 | ** | 27.49 | 67.96 | ** | 9.38 | 26.90 | ** | 11.54 | 23.46 | ** | 2.74 | 16.1 | ** | 3.55 | 34.8 | ** | 7.83 | 43.65 | ** | 0.31 | 17.22 | ** |
| *Oporornis tolmiei* |  |  |  | 16.0 | 43.43 | ** | 7.00 | 34.98 | ** | 16.63 | 46.61 | ** | 22.71 | 48.77 | ** | 51.06 | 86.7 | ** | 32.95 | 45.67 | ** | 8.23 | 26.9 | ** |
| *Oreothlypis celata* | 7.83 | 43.65 | ** | 3.55 | 34.8 | ** | 0.56 | 23.5 | ** | 0.45 | 5.78 | ** | 10.10 | 54.2 | ** | 0.19 | 6.03 | ** | 2.16 | 34.5 | ** | 20.99 | 40.63 | ** |
| *Oreothlypis ruficapilla* | 0.38 | 7.26 | * | 2.83 | 37.85 | ** | 2.38 | 21.88 | ** | 38.86 | 72.98 | ** | 33.97 | 69.95 | ** | 27.5 | 67.96 | ** | 37.01 | 58.29 | ** | 6.46 | 21.54 | ** |
| *Passerina ciris* |  |  |  | 7.18 | 28.5 | ** | 7.52 | 40.89 | ** | 13.49 | 48.73 | ** | 20.99 | 40.63 | ** | 57.87 | 98.43 | ** | 66.36 | 101.45 | ** | 9.38 | 26.9 | ** |
| *Passerina cyanea* |  |  |  | 11.54 | 23.46 | ** | 19.57 | 45.89 | ** | 19.15 | 57.67 | ** | 57.07 | 89.59 | ** | 23.78 | 80.76 | ** | 17.95 | 56.56 | ** | 25.19 | 70.97 | ** |
| *Piranga ludoviciana* |  |  |  | 0.31 | 17.22 | ** | 0.33 | 7.44 | * | 0.66 | 18.77 | * | 10.72 | 30.42 | ** | 4.08 | 20.92 | ** | 17.73 | 17.72 | ** | 0.99 | 14.77 | ** |
| *Setophaga citrina* | 0.65 | 18.77 | * | 27.5 | 67.96 | ** | 66.36 | 153.46 | ** | 32.95 | 45.67 | ** | 11.54 | 23.46 | ** | 1.56 | 22.42 | ** | 10.1 | 54.2 | ** | 11.54 | 23.46 | ** |
| *Setophaga magnolia* |  |  |  | 16.64 | 46.61 | ** | 2.74 | 16.1 | ** | 17.95 | 56.56 | ** | 0.56 | 23.50 | ** | 32.95 | 45.67 | ** | 11.54 | 23.46 | ** | 0.56 | 23.5 | ** |
| *Setophaga nigrescens* | 2.97 | 15.06 | ** | 0.66 | 8.81 | * | 0.92 | 18.57 | ** | 7.83 | 43.55 | ** | 16.0 | 43.43 | ** | 16.64 | 46.61 | ** | 18.93 | 44.37 | ** | 0.20 | 5.09 | * |
| *Setophaga occidentalis* | 1.53 | 8.1 | * | 10.1 | 54.2 | ** | 0.15 | 6.29 | * | 0.43 | 8.97 | * | 2.16 | 34.50 | ** | 0.09 | 9.13 | * | 0.56 | 23.5 | ** | 0.38 | 4.61 | * |
| *Setophaga virens* |  |  |  | 0.17 | 5.84 | * | 0.19 | 5.82 | * | 1.56 | 22.42 | ** | 2.74 | 16.1 | ** | 0.15 | 5.94 | * | 0.31 | 17.22 | ** | 0.18 | 6.37 | ** |
| *Spizella pallida* |  |  |  | 0.19 | 6.03 | ** | 0.56 | 23.5 | ** | 7.83 | 43.65 | ** | 8.23 | 26.9 | ** | 7.0 | 34.98 | ** | 57.07 | 89.89 | ** | 4.08 | 20.92 | ** |

Appendix 3

| *Passerina ciris* | *Passerina cyanea* | *Setophaga citrina* | *Setophaga virens* |
| --- | --- | --- | --- |
| Month  CV Tmax  0  2  4  6  8  10  O  N  D  E  F  M  A | Month  CV Tmax  0  2  4  6  8  10  O  N  D  E  F  M  A | Month  CV Tmax  0  2  4  6  8  10  O  N  D  E  F  M  A | Month  CV Tmax  0  2  4  6  8  10  O  N  D  E  F  M  A |
| Month  CV Tmin  0  5  10  15  20  O  N  D  E  F  M  A | Month  CV Tmin  0  5  10  15  20  25  O  N  D  E  F  M  A | Month  CV Tmin  0  5  10  15  S  O  N  D  E  F  M  A | Month  CV Tmin  0  5  10  15  20  O  N  D  E  F  M  A |
| Month  CV Prec  0  20  40  60  80  100  O  N  D  E  F  M  A | Month  CV Prec  0  20  40  60  80  100  120  O  N  D  E  F  M  A | Month  CV Prec  0  20  40  60  80  100  S  O  N  D  E  F  M  A | Month  CV Prec  0  20  40  60  80  100  O  N  D  E  F  M  A  Appendix 4 |

| *Oreothlypis celata* | *Oporornis tolmiei* | *Spizella pallida* | *Setophaga nigrescens* | *Setophaga occidentalis* | *Cardellina pusilla* |
| --- | --- | --- | --- | --- | --- |
| Month  CV Tmax  0  5  10  15  20  25  S  O  N  D  E  F  M  A | Month  CV Tmax  0  5  10  15  20  O  N  D  E  F  M  A | Month  CV Tmax  0  5  10  15  O  N  D  E  F  M  A | Month  CV Tmax  0  5  10  15  S  O  N  D  E  F  M  A | Month  CV Tmax  0  5  10  15  20  S  O  N  D  E  F  M  A | Month  CV Tmax  0  5  10  15  S  O  N  D  E  F  M  A |
| Month  CV Tmin  0  20  40  60  80  S  O  N  D  E  F  M  A | Month  CV Tmin  0  10  20  30  40  50  O  N  D  E  F  M  A | Month  CV Tmin  0  10  20  30  40  50  60  O  N  D  E  F  M  A | Month  CV Tmin  0  10  20  30  40  50  S  O  N  D  E  F  M  A | Month  CV Tmin  0  10  20  30  40  50  60  S  O  N  D  E  F  M  A | Month  CV Tmin  0  10  20  30  40  S  O  N  D  E  F  M  A |
| Month  CV Prec  0  20  40  60  80  100  S  O  N  D  E  F  M  A | Month  CV Prec  0  20  40  60  80  100  120  O  N  D  E  F  M  A | Month  CV Prec  0  20  40  60  80  100  O  N  D  E  F  M  A | Month  CV Prec  0  20  40  60  80  100  S  O  N  D  E  F  M  A | Month  CV Prec  0  20  40  60  80  100  120  S  O  N  D  E  F  M  A | Month  CV Prec  0  20  40  60  80  100  120  S  O  N  D  E  F  M  A |

.
